# A Smart Web Aid for Preventing Diabetes in Rural China: Preliminary Findings and Lessons

**DOI:** 10.2196/jmir.3228

**Published:** 2014-04-01

**Authors:** Penglai Chen, Jing Chai, Jing Cheng, Kaichun Li, Shaoyu Xie, Han Liang, Xingrong Shen, Rui Feng, Debin Wang

**Affiliations:** ^1^School of Health Services ManagementAnhui Medical UniversityHefeiChina; ^2^Lu’an Center for Diseases Prevention and ControlLu'anChina

**Keywords:** diabetes mellitus, prediabetic state, Internet, prevention, evaluation, eHealth

## Abstract

**Background:**

Increasing cases of diabetes, a general lack of routinely operational prevention, and a long history of separating disease prevention and treatment call for immediate engagement of frontier clinicians. This applies especially to village doctors who work in rural China where the majority of the nation’s vast population lives.

**Objective:**

This study aims to develop and test an online Smart Web Aid for Preventing Type 2 Diabetes (SWAP-DM2) capable of addressing major barriers to applying proven interventions and integrating diabetes prevention into routine medical care.

**Methods:**

Development of SWAP-DM2 used evolutionary prototyping. The design of the initial system was followed by refinement cycles featuring dynamic interaction between development of practical and effective standardized operation procedures (SOPs) for diabetes prevention and Web-based assistance for implementing the SOPs. The resulting SOPs incorporated proven diabetes prevention practices in a synergetic way. SWAP-DM2 provided support to village doctors ranging from simple educational webpages and record maintenance to relatively sophisticated risk scoring and personalized counseling. Evaluation of SWAP-DM2 used data collected at baseline and 6-month follow-up assessment: (1) audio recordings of service encounters; (2) structured exit surveys of patients’ knowledge, self-efficacy, and satisfaction; (3) measurement of fasting glucose, body mass index, and blood pressure; and (4) qualitative interviews with doctors and patients. Data analysis included (1) descriptive statistics of patients who received SWAP-DM2–assisted prevention and those newly diagnosed with prediabetes and diabetes; (2) comparison of the variables assessed between baseline and follow-up assessment; and (3) narratives of qualitative data.

**Results:**

The 17 participating village doctors identified 2219 patients with elevated diabetes risk. Of these, 84.85% (1885/2219) consented to a fasting glucose test with 1022 new prediabetes and 113 new diabetes diagnoses made within 6 months. The prediabetic patients showed substantial improvement from baseline to 6-month follow-up in vegetable intake (17.0%, 43/253 vs 88.7%, 205/231), calorie intake (1.6%, 4/253 vs 71.4%, 165/231), leisure-time exercises (6.3%, 16/253 vs 21.2%, 49/231), body weight (mean 62.12 kg, SD 9.85 vs mean 58.33 kg, SD 9.18), and body mass index (mean 24.80 kg/m^2^, SD 3.21 vs mean 23.36 kg/m^2^, SD 2.95). The prediabetic patients showed improvement in self-efficacy for modifying diet (mean 5.31, SD 2.81 vs mean 8.53, SD 2.25), increasing physical activities (mean 4.52, SD 3.35 vs mean 8.06, SD 2.38), engaging relatives (mean 3.93, SD 3.54 vs mean 6.93, SD 2.67), and knowledge about diabetes and risks of an imbalanced diet and inadequate physical activity. Most participating doctors and patients viewed SWAP-DM2 as useful and effective.

**Conclusions:**

SWAP-DM2 is helpful to village doctors, acceptable to patients, and effective in modifying immediate determinants of diabetes at least in the short term, and may provide a useful solution to the general lack of participation in diabetes prevention by frontier clinicians in rural China.

**Trial Registration:**

International Standard Randomized Controlled Trial Number (ISRCTN): 66772711; http://www.controlled-trials.com/ISRCTN66772711 (Archived by WebCite at http://www.webcitation.org/6OMkAqyEy).

## Introduction

The rates of type 2 diabetes mellitus (DM2) are increasing and it is expanding rapidly from primarily affecting people in developed nations to also afflicting people in the developing world [1-3]. It is predicted that diabetes will claim up to 366 million people worldwide by 2030 [4]. This epidemic is also growing in China [5-7]. A recent nationwide investigation revealed that age-standardized prevalence of total diabetes in China is as high as 11.4% in urban areas and 8.2% in rural areas [8-10]. Diabetes interacts with other major risk factors (eg, hypertension, dyslipidemia) and increases the risk of a variety of morbidities (eg, chronic kidney disease, end-stage renal disease, atherosclerosis, coronary heart disease, and cerebral ischemia) leading to tremendous physical, psychological, and socioeconomic suffering and burden [11-13]. Diabetes develops from prediabetes, a lesser degree of hyperglycemia [14,15], and it is well established that the risk of this progression can be modified substantially (by 20% to 60%) regardless of nation and population group [16-20]. Encouraged by high efficacies, research efforts have been invested in this regard and a whole range of information, education, and communication are available, including guidelines, protocols, tool kits, and best practices [21,22].

Unfortunately, proven interventions against diabetes are not being practiced routinely in China. This is especially true in the resource-poor rural areas where more than 75% of the vast population lives. Previously published studies and our own preliminary investigations all suggest that rural village doctors seldom participate in identifying and preventing diabetes [23,24]. As a result, 42% to 82% of rural villagers newly screened with diabetes had never been diagnosed with the disease before and knowledge about prediabetes or diabetes among rural villagers is extremely low. The primary reason for this gap between proven technologies and application may relate to a general lack of necessary knowledge and skills among village doctors in rural China. Less than 12% of village doctors have received formal training on diabetes, and only 43% knew basic knowledge about diabetes prevention [25,26]. Another barrier preventing routinely implementing proven interventions may be lack of incentives [27,28]. Persistent promotion of lifestyle modification, the key to diabetes prevention, requires sustained momentum. This calls for continuous monitoring, supervising, and rewarding. However, all these are generally missing with the current health care systems in China, especially those in resource-poor areas.

In response to these challenges and others, we started a quasi-cluster randomized controlled trial (ISRCTN66772711) in Lu’an, Anhui, China. It aimed at devising, implementing, and evaluating an intervention package to reduce progression from prediabetes to diabetes among male and female farmers aged 40 and older and establishing a sustainable mechanism for integrating diabetes prevention within routine medical service. The trial utilized a batched implementation strategy in which villages were recruited in blocks to practice planned intervention with former blocks informing later ones. For each block, measurement occured at baseline and every 12 months (for plasma glucose) or monthly (for knowledge, behavior, body weight, and blood pressure) after baseline. The intervention package is known as educating doctors and electronic supports, counseling diabetes prevention, recipe for lifestyle modification, operational toolkit, performance-based reimbursement for doctors, and screening service (eCROPS). The overall trial protocol has been published elsewhere [29]. This paper documents the “e” component of the intervention package. The Smart Web Aid for Preventing Type 2 Diabetes (SWAP-DM2) was designed to tackle existing barriers to applying proven interventions and integrating diabetes prevention with routine medical care by using evidence-based measures in an innovative and synergetic way.

## Methods

### Development of Initial SWAP-DM2

The SWAP-DM2 inherited the strategies and theoretical frameworks we used successfully in producing a practical computerized expert system for routine human immunodeficiency virus (HIV) voluntary counseling and testing that simplified the counseling process and leveraged essential procedures and best practices [30]. It utilized Microsoft Visual Studio 2008 as the platform, SQL Server 2008 as the data management tool, C# as the programming language, and evolutionary prototyping as the overall development approach in which design of the initial type system was followed by continuous refinement cycles. The whole process featured dynamic interaction between development of practical and effective standardized operation procedures (SOPs) and Web-based assistance for implementing these SOPs.

The initial SOPs behind SWAP-DM2 were derived through evidence- and theory-based consensus. First, our research team on diabetes prevention conducted a systematic literature review of related guidelines, protocols, theories, and research articles and then worked out a long list of proven intervention approaches and models. Second, an expert panel consisting of experienced counselors, psychologists, clinicians, and nurses who work with patients with diabetes, as well as experts on nutrition and physical activities and epidemiologists conveyed for a consensus meeting and produced a short list of proven interventions from the long list through clarification, brainstorming, and rounds of voting. Third, our technical groups on diabetes prevention and Web program development worked together and translated the short list of intervention approaches and models into primitive SOPs. Fourth, the expert panel gathered again and revised the primitive SOPs into initial practical ones. Motivational interviewing played a guiding role in this process because the core part of the SOP development concerned counseling lifestyle management and the theory has been applied successfully for promoting behavior changes in various population groups (Multimedia Appendix 1) [31].

The prototype SWAP-DM2 built upon the previously mentioned SOPs through close collaboration between software programmers and diabetes prevention researchers within our team. First, our diabetes prevention researchers clarified on an item-by-item basis the SOPs to our Web programmers and addressed all enquiries raised by the latter. Following this, the same 2 groups continued working together and performed an individual needs analysis in which each item of the SOPs underwent a semistructured process for identifying potential Web-based electronic supports for implementing the SOP by using nominal group techniques [32,33]. Then, the programmers grouped and combined all the needs identified into pragmatic applications and program codes.

### SWAP-DM2: Mediated Diabetes Prevention

**Figure 1:**
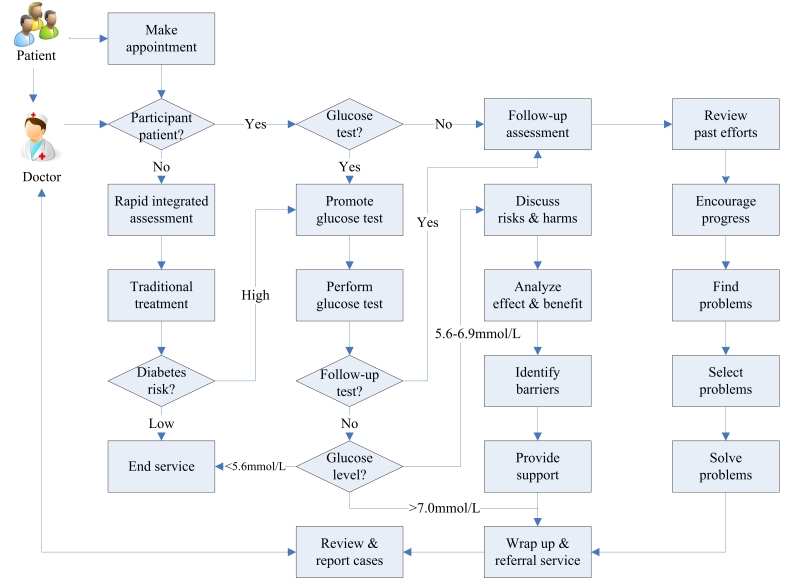
Flowchart of the Smart Web Aid for preventing type 2 diabetes (SWAP-DM2).

As summarized in Multimedia Appendix 2, the SWAP-DM2 provides a package of support for village doctors in delivering diabetes prevention ranging from simple educational webpages and record maintenance to relatively sophisticated risk scoring and personalized counseling. The essence of the Web is that it leverages the use of SOPs and best diabetes prevention in a user-friendly way. The users (village doctors in this case) need to mainly follow the steps proposed and make minimum responses by either clicking the mouse or pressing an access key depending on the users’ preferences. Figure 1 depicts the general process of SWAP-DM2–mediated prevention and Multimedia Appendix 3 provides sample application webpages.

For a given patient presenting to a clinic, SWAP-DM2 automatically classifies (after inputting a unique identification number) the patient as participant or nonparticipant of the diabetes prevention project and then proposes standard service for each kind of patient accordingly. If the patient was a nonparticipant, the system provided SOPs for performing an integrated rapid assessment (ie, assessing the patient’s risk for developing diabetes by using a very short instrument while the patient received traditional medical service), which in turn enabled the system to automatically assign the patient to either a high- or low-risk nonparticipant patient group. For a high-risk nonparticipant patient, SWAP-DM2 lead to standard procedures for promoting and performing a glucose test, which then allowed for further classification of the patient as suspected diabetes (fasting glucose ≥7.0 mmol/L), prediabetes (fasting glucose 5.6-6.9 mmol/L), or normal (fasting glucose <5.6 mmol/L). For a normal patient, SWAP-DM2 tells the doctor to end the service for the patient. For suspected diabetes, the system suggests an SOP referring the patient to higher-level diabetes diagnosis and treatment services. As for prediabetes, SWAP-DM2 proposes a 4-step SOP for promoting the patient to participate in consecutive cycles of lifestyle management. These 4 steps are (1) discussing risks to the patient and the harms diabetes causes, (2) analyzing effectiveness and benefits of lifestyle management, (3) identifying barriers to lifestyle management, and (4) providing supports for overcoming the barriers. Each time a participant patient presented, SWAP-DM2 mediated a tailored round of lifestyle management. Each round of lifestyle management comprised 6 core steps (ie, perform follow-up assessment, review previous efforts, encourage progress, identify problems, select problems, and solve problems) for all participants and 2 additional steps (ie, promote and perform glucose test) for selective patients. *Tailored* here means that detailed content of each round of lifestyle management differed from others and depended on the patient’s previous involvement in and performance on lifestyle management.

### SWAP-DM2 Assessment Process and Measures

The initial SWAP-DM2 described previously has been refined through repeated cycles of testing and assessment. The pilot work started in early June 2013 and the assessment occurred at baseline (in the week before training of participating village doctors) and every month after SWAP-DM2 application (in the last week of the month). Village doctors from randomly selected clinics in rural Lu’an (one of the largest prefectures in Anhui province) were invited to participate in testing the system.

Assessment is an integral part of the refinement of SWAP-DM2. This study did not collect any additional information; rather, it utilized data generated during the baseline and the 6-month follow-up assessment of SWAP-DM2 application scheduled during the last week of the month. These assessments consisted of (1) prevention case recording, in which all the patient-doctor encounter episodes happened at the participating clinics during the selected week were, after informed consent, audio recorded; (2) exit survey using a structured questionnaire (ie, after completion of each service during the assessment week), the eligible patient (aged 40 to 70 years and tested as prediabetics) was led to a private room and interviewed by a trained interviewer using a structured questionnaire to solicit information about his/her knowledge about diabetes and self-efficacy (self-perceived ability) to practice indicative lifestyle modification against diabetes and satisfaction toward the service just received (Multimedia Appendix 4); (3) measurements including body height and weight, fasting glucose (baseline and annual follow-up evaluations), and blood pressure; and (4) qualitative interview (follow-up evaluation only) in which all participating village doctors and 1-2 patients from each doctor’s clinic were interviewed by trained researchers using a checklist of open-ended questions about SWAP-DM2 (eg, its effects on diabetes intervention, areas to improve, barriers to utilization). The exit survey questionnaire covered all key aspects, according to the motivational interviewing theory mentioned previously, of diabetes-related lifestyle management (see Multimedia Appendix 3). It tested moderately reliable with a standardized Cronbach alpha of .74, .85, .70, .77, and .98 for knowledge about diabetes and prediabetes, harms of inadequate physical activity and imbalanced diet, self-efficacy, diabetes-related behaviors, and satisfaction toward Web aid-supported service, respectively.

Data was collected using both quantitative and qualitative methods. The audio-recorded cases of service delivery were used to generate quantitative service quality data through (1) translating the audio recordings into textual transcripts; (2) mixing the baseline case transcripts with the follow-up ones with all identification information being removed except for a unique number to ensure blind assessment; (3) sending the mixed transcripts to 2 researchers and asking them check each of the cases independently against a list of essential prevention procedures and judge whether these essential prevention procedures had been delivered; and (4) having the 2 researchers sit together and reach consensus over their disagreements.

### Data Analysis

Data analysis used SPSS 16.0 (SPSS Inc, Chicago, IL, USA) and included (1) descriptive numbers and percentages of patients who received SWAP-DM2–assisted prevention and newly diagnosed prediabetes and diabetes; and (2) comparison, using chi-square tests for percentages or *t* tests for quantitative ratings, between the baseline and 6-month follow-up assessments in terms of improvements in delivery of essential prevention procedures, knowledge about diabetes, lifestyle management practices, body mass index (BMI), and blood pressure, etc; and (3) narratives of qualitative data.

### Human Subject Protection

This study involves recruitment, intervention, and assessment of villagers and village doctors. So it adheres to rigorous human subject protection principles and procedures. The study protocol had been reviewed and approved by the Biomedical Ethics Committee of Anhui Medical University. Participation of villagers and village doctors was 100% voluntary. Written informed consent was sought from all participants.

## Results

### Patient and Doctor Participants

A total of 17 village doctors participated in pilot-testing SWAP-DM2 and they identified 2219 patients at risk for being diagnosed with prediabetes or diabetes using the rapid assessment function of the system during this preliminary evaluation period. Of these 2219 patients, 1885 (85.94%) received a free fasting glucose test; 1022 (54.22%) and 103 (5.46%), were diagnosed with prediabetes and diabetes, respectively. Most patients diagnosed with prediabetes (95.3%, 974/1022) did not know their glucose status before (Table 1).

The total number of patients encountered and identified as having prediabetes during baseline and 6-month follow-up assessment were 253 and 231, respectively. The intervention duration of the follow-up assessment for participants ranged from 1 month to 6 months. No statistically significant differences in gender, age, and education level were found between these 2 groups. Approximately two-thirds of these participating patients were females, 171 (67.59%) and 160 (69.26%) for the baseline and follow-up groups, respectively.

**Table 1:** Description of patients assessed by the Smart Web Aid for Preventing Type 2 Diabetes (SWAP-DM2).

Service output	Total	Gender		Age (years)^a^		
		Male	Female	≥50	51-60	61-70
Patients assessed with elevated prediabetes risk,^b^ n	2219	807	1412	825	590	804
Patients took fasting glucose test, n (%)	1885 (84.94)	675 (83.64)	1210 (85.69)	679 (82.30)	515 (87.28)	691 (85.95)
Patients tested with prediabetes, n (%)	1022 (54.22)	348 (51.56)	674 (55.70)	339 (49.93)	261 (50.68)	422 (61.07)
Number of newly diagnosed prediabetes, n (%)	974 (95.30)	333 (95.69)	641 (95.10)	327 (96.46)	249 (95.40)	398 (94.31)
Number of newly diagnosed diabetes	103 (5.46)	39 (5.78)	64 (5.29)	39 (5.74)	26 (5.05)	38 (5.50)

^a^Age ranges are approximate.

^b^Prediabetes denotes fasting glucose ≥5.6 mmol/L and <7.0 mmol/L.

### Delivery of Essential Prevention Procedures

Before the SWAP-DM2 application, the proportion of patients who received the essential prevention procedures assessed was extremely low. The most commonly delivered essential prevention procedure was measuring blood pressure, accounting for 43.9% (111/253) of the patients, followed by assessing diet behavior (17.0%, 43/253), and physical activity (13.0%, 33/253). Compared with baseline, SWAP-DM2 utilization significantly increased delivery of almost all essential prevention procedures listed in Table 2 except for glucose measurement, which decreased from 9.1% (23/231) to 1.3% (3/231). Assessing diet behavior (17.0%, 43/253 to 100.0%, 231/231), physical activity (13.0%, 33/253 to 95.7%, 221/231), and BMI (10.7%, 27/253 to 100%, 231/231) witnessed the greatest improvement, followed by counseling barriers to (0.8%, 2/253 vs 81.4%, 188/231) and skills for (3.2%, 8/253 vs 90.9%, 210/231) modifying diet. No statistically significant difference was observed in delivery of the essential prevention procedures at baseline or follow-up between male and female patients.

**Table 2:** Essential prevention procedures delivered in traditional and SWAP-DM2–mediated service.

Essential prevention procedures	Male, n (%)			Female, n (%)			Total, n (%)		
	Baseline (n=82)	Follow-up (n=71)	*P*	Baseline (n=171)	Follow-up (n=160)	*P*	Baseline (n=253)	Follow-up (n=231)	*P*
Assessing diet behavior	16 (19.5)	71 (100)	<.001	27 (15.8)	160 (100)	<.001	43 (17.0)	231 (100)	<.001
Assessing physical activity	17 (20.7)	70 (98.6)	<.001	16 (9.4)	151 (94.4)	<.001	33 (13.0)	221 (95.7)	<.001
Measuring body mass index	9 (11)	71 (100)	<.001	18 (10.5)	160 (100)	<.001	27 (10.7)	231 (100)	<.001
Measuring blood pressure	33 (40.2)	64 (90.1)	<.001	78 (45.6)	150 (93.8)	<.001	111 (43.9)	214 (92.6)	<.001
Measuring blood glucose	7 (8.5)	0 (0)	.89	16 (9.4)	3 (1.9)	.54	23 (9.1)	3 (1.3)	.54
Counseling diabetes susceptibility	0 (0)	48 (67.6)	<.001	3 (1.8)	114 (71.3)	<.001	3 (1.2)	162 (70.1)	<.001
Counseling risks of unhealthy diet	5 (6.1)	43 (60.6)	<.001	10 (5.8)	96 (60.0)	<.001	15 (5.9)	139 (60.2)	<.001
Counseling risks of inadequate activity	4 (4.9)	42 (59.2)	<.001	8 (4.7)	94 (58.8)	<.001	12 (4.7)	136 (58.9)	<.001
Counseling barriers to modifying diet	1 (1.2)	58 (81.7)	<.001	1 (0.6)	130 (81.3)	<.001	2 (0.8)	188 (81.4)	<.001
Counseling barriers to increasing activity	0 (0)	49 (69.0)	<.001	1 (0.6)	112 (70.0)	<.001	1 (0.4)	161 (69.7)	<.001
Counseling skills to modify diet	0 (0)	63 (88.7)	<.001	8 (4.7)	147 (91.9)	<.001	8 (3.2)	210 (90.9)	<.001
Counseling skills to increase activity	0 (0)	59 (83.1)	<.001	3 (1.8)	137 (85.6)	<.001	3 (1.2)	196 (84.9)	<.001

### Changes in Patients’ Immediate Indicators

As shown in Table 3, SWAP-DM2 helped to raise the prediabetic patients’ knowledge about the risks of diabetes, imbalanced diet, and inadequate physical activity. It also worked well in improving the patients’ self-efficacy in eating a healthier diet and increasing physical activities. Most importantly, SWAP-DM2–mediated prevention substantially improved key diabetes-related behaviors among the prediabetic patients, with the greatest improvement observed in increasing vegetable intake and a relatively smaller change for increasing exercise. Body weight and BMI of the prediabetic patients also showed statistically significant improvement. The patients’ satisfaction toward the service as a whole and their doctor’s responsiveness remained unchanged, whereas their satisfaction toward the service technology increased slightly. Most of these changes showed no statistically significant differences between gender groups. Self-efficacy for increasing physical activity and engaging relatives, however, had greater improvement in males than females, mean 8.64 (SD 2.31) vs mean 7.80 (SD 2.41, *P*=.01) and mean 7.80 (SD 2.41) vs mean 6.54 (2.70, *P*<.001), respectively.

### Patients’ and Doctors’ Views Toward SWAP-DM2

All village doctors who tested the SWAP-DM2 and a convenience sample of 20 prediabetic patients who experienced the Web-facilitated diabetes prevention service participated in our qualitative interviews. The doctors viewed SWAP-DM2 as helpful and easy:

It’s an innovative and real expert system.

It standardizes diabetes prevention and proposes pertinent procedures and information.

With the Web, identifying high-risk patients and counseling lifestyle modification becomes easy.

By following its steps, you won’t miss important things.

It helps communicate balanced information comprising not only benefits but also “dis-benefits,” not only barriers but also measures to overcome barriers; and this is especially helpful in motivating sustainable lifestyle changes.

Perhaps the biggest advantage is its ability in tailoring prevention to individual needs and bridging current and past services into continuous prevention.

The doctors also raised several concerns about SWAP-DM2:

Although desktop computers and Internet are available at most village clinics, electricity supply is not totally secured.

The Web adopts a step-by-step approach in counseling lifestyle changes; patients’ responses may go “awry” or may not quite fit the proposed procedures occasionally and this means doctors should have enough experience in leading the communication.

The Web system does not work so well during outreach, although it is accessible via mobile phones; mobile communication service charges are not refundable by government or health insurance agencies for most village doctors at present.

The patients’ views toward SWAP-DM2–assisted prevention were also mostly positive:

A Web aid plus a doctor must be better than a doctor without any help; more importantly, it is a smart Web designed by experienced experts with a credited medical college.

I had never thought of taking glucose test before. Yet I felt most necessary to do so when my doctor told me, after having asked a few questions according to the screen and entered my responses that I was at increased risk for diagnosing glucose impairment since...

I felt pressing when my doctor pointed to the screen and said, “Your body weight keeps going up and let’s decide on something about it.”

At each follow-up visit, my doctor always refers to the Web and says, “Let’s see what we had decided to do last time,” this reminds me repeatedly that the Web has a good memory and I should keep my words.

With regard to concerns that computer operation may depersonalize service provision, most patients disagreed:

I am used to computer-aided services; it happens almost everywhere like supermarkets, banks and so on.

I did not feel any uneasiness.

However, some patients mentioned “my doctor referred to a computer from time to time, it was more or less different from a free discussion” or “being an old and illiterate farmer, I do not know how the Web had helped me.”

**Table 3:** Patients’ knowledge, self-efficacy, satisfaction, and behavioral outcome measures.

Variable assessed		Male			Female			Total		
		Baseline (n=82)	Follow-up (n=71)	*P*	Baseline (n=171)	Follow-up (n=160)	*P*	Baseline (N=253)	Follow-up (N=231)	*P*
**Knowledge about risks of diabetes/prediabetes, n (%)**										
	Leads to eye, kidney, heart lesions	2 (2.4)	55 (77.5)	<.001	5 (2.9)	129 (80.6)	<.001	7 (2.8)	184 (79.7)	<.001
	Affects long-term objectives and development	5 (6.1)	39 (54.9)	<.001	7 (4.1)	83 (51.9)	<.001	12 (4.7)	122 (52.8)	<.001
	Affects family and social relationships	3 (3.7)	31 (43.7)	<.001	3 (1.8)	60 (37.5)	<.001	6 (2.4)	91 (39.4)	<.001
	Induces psychological and economic burdens	3 (3.7)	42 (59.2)	<.001	2 (1.2)	77 (48.1)	<.001	5 (2.0)	119 (51.5)	<.001
**Knowledge about risks of imbalanced diet, n (%)**										
	Leads to overweight or obesity	2 (2.4)	43 (60.6)	<.001	8 (4.7)	108 (67.5)	<.001	10 (4.0)	151 (65.4)	<.001
	Causes hypertension	10 (12.2)	60 (84.5)	<.001	6 (3.5)	118 (73.8)	<.001	16 (6.3)	178 (77.1)	<.001
	Leads to cerebral and cardiovascular diseases	4 (4.9)	33 (46.5)	<.001	3 (1.8)	62 (38.8)	<.001	7 (2.8)	95 (41.1)	<.001
	Induces diabetes	5 (6.1)	49 (69)	<.001	7 (4.1)	102 (63.8)	<.001	12 (4.7)	151 (65.4)	<.001
	Leads to cancer	0 (0.0)	19 (26.8)	<.001	3 (1.8)	56 (35.0)	<.001	3 (1.2)	75 (32.5)	<.001
**Knowledge about risks of inadequate physical activity, n (%)**										
	Leads to overweight or obesity	5 (6.1)	63 (88.7)	<.001	15 (8.8)	151 (94.4)	<.001	20 (7.9)	214 (92.6)	<.001
	Causes hypertension	4 (4.9)	53 (74.7)	<.001	8 (4.7)	120 (75.0)	<.001	12 (4.7)	173 (74.9)	<.001
	Leads to cerebral and cardiovascular diseases	3 (3.7)	31 (43.7)	<.001	5 (2.9)	66 (41.3)	<.001	8 (3.2)	97 (42.0)	<.001
	Induces diabetes	5 (6.1)	34 (47.9)	<.001	4 (2.3)	65 (40.6)	<.001	9 (3.6)	99 (42.9)	<.001
	Leads to cancer	1 (1.2)	28 (39.4)	<.001	0 (0.0)	45 (28.1)	<.001	1 (0.4)	73 (31.6)	<.001
	Reduces body immunity	2 (2.4)	36 (50.7)	<.001	5 (2.9)	84 (52.5)	<.001	7 (2.8)	120 (51.9)	<.001
**Self-efficacy ratings,** ^a^ **mean (SD)**										
	Modifying diet	5.38 (2.79)	8.59 (2.23)	<.001	5.28 (2.85)	8.50 (2.28)	<.001	5.31 (2.81)	8.53 (2.25)	<.001
	Increasing physical activities	4.85 (3.26)	8.64 (2.31)	<.001	4.36 (3.39)	7.80 (2.41)	<.001	4.52 (3.35)	8.06 (2.38)	<.001
	Refusing snacks	8.97 (2.00)	9.88 (1.88)	<.001	8.64 (2.29)	9.43 (2.15)	<.001	8.75 (2.20)	9.57 (2.08)	<.001
	Engaging relatives in diabetes prevention	4.41 (3.21)	7.80 (2.41)	<.001	3.70 (3.58)	6.54 (2.70)	<.001	3.93 (3.54)	6.93 (2.67)	<.001
**Behaviors practiced, n (%)**										
	Reduced calorie intake	2 (2.4)	55 (77.5)	<.001	2 (1.2)	110 (68.8)	<.001	4 (1.6)	165 (71.4)	<.001
	Increased vegetable intake	15 (18.3)	64 (90.1)	<.001	28 (16.4)	141 (88.1)	<.001	43 (17.0)	205 (88.7)	<.001
	Increased leisure-time exercises	5 (6.1)	15 (21.1)	<.001	11 (6.4)	34 (21.3)	<.001	16 (6.3)	49 (21.2)	<.001
**Immediate outcome measures, mean (SD)**										
	Body weight (kg)	67.31 (10.03)	63.68 (9.35	.023	59.63 (9.00)	55.95 (9.11)	<.001	62.12 (9.85)	58.33 (9.18)	<.001
	Body mass index (kg/m^2^)	24.32 (3.15)	23.01 (2.86)	.008	25.03 (3.32)	23.52 (2.97)	<.001	24.80 (3.21)	23.36 (2.95)	<.001
	Systolic blood pressure (mm Hg)	141.21 (21.07)	137.23 (19.56)	.23	135.57 (21.80)	131.54 (20.07)	.08	137.40 (21.50)	133.29 (19.80)	.03
	Diastolic blood pressure (mm Hg)	87.89 (13.63)	87.01 (12.89)	.68	84.4 (11.98)	82.92 (11.65)	.26	85.90 (12.67)	84.18 (12.02)	.13
**Satisfaction ratings,** ^a^ **mean (SD)**										
	Toward service provided	9.28 (1.53)	9.11 (1.48)	.49	8.82 (1.86)	8.73 (1.82)	.66	8.97 (1.77)	8.85 (1.73)	.45
	Toward service techniques used	8.86 (1.89)	9.20 (1.76)	.25	8.71 (1.94)	9.10 (1.78)	.06	8.76 (1.92)	9.13 (1.77)	.03
	Toward doctor’s responsiveness	9.49 (1.07)	9.40 (1.05)	.60	9.05 (1.54)	9.04 (1.52)	.95	9.20 (1.42)	9.15 (1.41)	.70

^a^Maximum=10.

## Discussion

### Effectiveness and Acceptability of SWAP-DM2

By using SWAP-DM2, 17 village doctors identified 2219 patients with elevated prediabetes and diabetes risk, encouraged 85.0% of them perform a fasting glucose test, and diagnosed 1022 prediabetes and 103 new diabetes cases within only 6 months. More importantly, these prediabetic patients showed substantial improvement from baseline to 6 months after intervention in terms of vegetable intake, calorie intake, leisure-time activities, and even body weight and BMI. The prediabetic patients also witnessed great improvements in self-efficacy in modifying diet, increasing physical activities, etc. These findings are consistent and point to an encouraging implication that SWAP-DM2 is effective, at least in the short term. The primary reason underlying this effectiveness may be that SWAP-DM2 incorporates, in a synergetic way, a variety of proven strategies and techniques. For example, it ensures delivery of essential prevention procedures (Table 3) through required SOPs, leverages behavior changes via convincing evidences, such as changes and trends in the patient’s own BMI, fasting glucose, etc, and facilitates counseling lifestyle modification by using motivational interviewing. In contrast to most other essential prevention procedures, glucose testing decreased from baseline to 6-month follow-up. This was because the SOPs required that the test occur only annually for patients with elevated risk. The nonsignificant changes in the patients’ satisfaction variables may be due primarily to the fact that the same satisfaction ratings at baseline were already quite high and thus room for improvement was limited.

This preliminary evaluation also revealed indications that SWAP-DM2 was acceptable to both villagers and village doctors. These included the positive comments solicited during the qualitative interviews and the frequent use of the Web as indicated by the large volume of SWAP-DM2–mediated risk assessments, glucose tests, and counseling sessions delivered during the short trial period, and the more than 88.5% satisfaction of the villagers toward the Web-assisted services. A number of factors may have contributed to this acceptance: (1) SWAP-DM2 transforms complex diabetes education or counseling into step-by-step procedures and makes it easy to learn and practice; (2) SWAP-DM2 enriches service content and procedures with little added workload by real-time data recording and retrieving, and automatic behavior risk identification, classification, and lifestyle modification planning; and (3) SWAP-DM2 gains credibility (for doctors) and confidence (in patients) by providing highly professional and tailored suggestions and by demonstrating high-tech (ie, Internet and expert system) use. There existed a sharp discrepancy between the number of female patients identified as being prediabetic and female participants in both the baseline and follow-up assessments than males. This may not necessarily indicate that the SWAP-DM2–aided prevention was more acceptable among females that males. Rather, it may be explained by the fact that most male villagers were pursuing temporary jobs in cities during the trial period.

### Limitations

First, this study documented only a segment of an ongoing project [29]. Although the overall design of this project belongs to a quasi-randomized controlled trial, findings presented in this paper allowed for only baseline vs follow-up rather than intervention vs control comparisons. Therefore, readers are cautioned about a variety of potential confounding factors in interpreting the findings (eg, national or regional diabetes education programs during the same period of this study). Second, the evaluation of SWAP-DM2 covered only 6 months of intervention and used mostly short-term variables. Whether the Web-mediated prevention continues to work smoothly in the long run when doctors and patients have lost curiosity about Web-based systems and whether the immediate indicators (eg, knowledge, self-efficacy, healthy lifestyle practices, BMI) ultimately lead to desired outcomes (eg, reduced diabetes progression) needs further investigation. Some previous studies showed that body weight, fasting glucose, etc, decreased from baseline to their lowest level at approximately 6 months after intervention and then began to rise again slowly [34]. Third, there may be some seasonal effects. The villagers may have different amounts of farm work to do in different seasons. Their food availability and eating and cooking habits may also change across seasons. This may result in quite different findings over the 6 months. Fourth, the SWAP-DM2-mediated prevention service (including glucose tests, blood and body weight measurements, etc) was provided free of charge to the villagers and the participating doctors were reimbursed, through a temporary project, for their time and efforts invested at a minimum rate (approximately US $5 per prevention case per year). This may raise sustainability concerns, although it would not be very difficult to get permanent government support if this prevention proves to be cost-effective. In addition, readers are reminded of the measurement limitations because most of the patient outcomes were derived from self-repot data that were prone to various biases (eg, social desirability and recency biases).

### Lessons From SWAP-DM2 Development and Application

Given the number of elements involved and the complex relationships between them, it is almost impossible to distinguish effects of any specific elements except the overall SWAP-DM2–supported intervention as a whole. Therefore, the key to development of similar systems and transferring lessons from this study to future interventions is leveraging smart Web applications in selecting and combining proven elements into an integrated package. Another strategy getting around the complexity just mentioned toward effectiveness is rapid prototyping followed by continuous refinement. This includes not only the application and assessment cycles as described in the methodology section, but also the inbuilt self-learning mechanisms with the Web-based system as shown in Multimedia Appendix 2 (eg, learned tailoring of risk assessment and behavior counseling). Projects of this kind depend heavily on effectively bridging 2 heterogeneous aspects of expertise (ie, diabetes prevention and information technology). Although repeated interaction between these 2 specialty groups helped in our case, the leading role of a diabetes prevention expert who has adequate knowledge of Web-based applications of modern information technologies seemed to be even more important. Finally, necessary structural changes should accompany application of soft-systems such as this. As introduced in the umbrella protocol of this study, a set of performance-based incentives were implemented alongside SWAP-DM2 application. These may have played some role in the short-term—yet encouraging—findings of this study. In addition, approaches for promoting application of the system also merits careful consideration. For instance, the village doctors were often misled by the seemingly complex workflows and functions as described in Figure 1 and Multimedia Appendix 2. They should only be used to provide readers with an overview of what SWAP-DM2 can do in overall rather than what it does in a single encounter. A typical SWAP-DM2–assisted intervention session involves only part of the steps and takes 10 to 40 minutes depending on the patient’s needs. Although the system “behind” the interface is quite sophisticated, the Web pages were designed to be as smooth and simple as possible. Therefore, stepwise learning by doing may be an effective way to gain and train village doctors.

### Conclusions

Our preliminary findings suggest that SWAP-DM2 is helpful to village doctors, acceptable to patients, and effective in modifying immediate determinants of diabetes at least in the short term. Rapidly growing diabetes cases contrasted by a general lack of routinely operational prevention against the epidemic calls for immediate engagement of frontier clinicians, especially village doctors who work in rural China where the majority of the nation’s vast population lives [35-37]. Given the long history of separating disease prevention and treatment and the extremely limited resources in rural China, this depends heavily on effectively tackling a series of barriers [38,39]. SWAP-DM2 may provide a useful solution in reaching this end.

## Multimedia Appendix 1

Guiding Models and principles for developing standardized operation procedures (SOPs) of counseling diabetes prevention.

## Multimedia Appendix 2

Main functions of Smart Web Aid for Preventing Diabetes (SWAP-DM2).

## Multimedia Appendix 3

Sample application webpages of Smart Web Aid for Preventing Type 2 Diabetes (SWAP-DM2).

## Multimedia Appendix 4

Exit survey for Smart Web Aid for Preventing Type 2 Diabetes (SWAP-DM2) assessment.

## Abbreviations

BMI: body mass index
DM2: type 2 diabetes mellitus
eCROPS: educating doctors and electronic supports, counseling diabetes prevention, recipe for lifestyle management, operational toolkit, performance-based reimbursement for doctors, and screening service
SOP: standardized operation procedure
SWAP-DM2: Smart Web Aid for Preventing Type 2 Diabetes
