# Supplementary material for: A Smart Web Aid for Preventing Diabetes in Rural China: Preliminary Findings and Lessons
Source: J Med Internet Res. 2014 Apr 1;16(4):e98. doi: 10.2196/jmir.3228 (PMC4004141; doi:10.2196/jmir.3228)
Supplement: Supplementary file 1 [file jmir_v16i4e98_app1.pdf]

**Appendix 1 Guiding Models and principles for developing standardized operation procedures (SOPs) of counseling diabetes prevention.**

**A. Theoretical Underpinnings**

eCROPS diabetes prevention counseling (DPC) is based on motivational interviewing. It draws from social psychology, including theories of cognitive dissonance and self-efficacy, as well as empathic processes and views motivation as a state of readiness for change rather than a personality trait that is relatively stable. Lack of motivation (or resistance to change), therefore, is not a set individual characteristic but rather malleable. When an individual's motivation is mixed, we call this ambivalence, which is often voiced through the expression of both the positive and negative aspects of the behavior. Motivational interviewing is a client-centered, personalized and directive style of counseling designed to assist individuals in resolving ambivalence and increasing their commitment to change. The counselor-counselee relationship is more like a partnership here than it is in more traditional expert/recipient models. The counselor respects the participant's autonomy and freedom of choice (and consequences) regarding his/her own behavior.

**Cognitive dissonance**

- Cognitive dissonance is the feeling of psychological discomfort produced by the combined presence of two thoughts that do not follow from one another;
- Being psychologically uncomfortable, the existence of dissonance motivates the person to reduce the dissonance and leads to avoidance of information likely to increase the dissonance; the greater the discomfort is, the greater the desire to reduce the dissonance of the two cognitive elements;
- Motivational interviewing produces a dissonant state (by focusing on ambivalence or inconsistencies) and then controls the direction chosen for the dissonance resolution through the skilled use of motivational interviewing techniques;
- Ambivalence is not a barrier but a crucial entry point and can be resolved by focusing on the participant's desires, expectations, beliefs, fears, and hopes, with particular emphasis on the inconsistencies between these and the problematic behavior.

**Self-efficacy:**

- Self-efficacy is a person’s belief that he/she can carry out —and succeed at —a specific change strategy;
- People with high efficacy expect to succeed, realize favorable outcomes and

vice versa;

- Self-efficacy beliefs also determine how obstacles and impediments are viewed;
- People with high efficacy believe that they can overcome obstacles by persevering and by improving self-management skills and they do not give up, but rather "stay the course" in the face of difficulties;
- People with low efficacy believe that their efforts in the face of difficulties will fail and would therefore be a waste of time to undertake and they quickly give up trying.

**Accurate Empathy:**

- Accurate empathy defines skillful reflective listening that clarifies and amplifies the participant's own experience and meaning, without imposing the counselor’s own material;
- It is unlikely to evoke participant resistance;
- It encourages the participant to keep talking and exploring the topic;
- It communicates respect and caring, and builds a working alliance between counselor and participant;
- It clarifies for the counselor exactly what the participant means;
- It can be used to reinforce ideas expressed by the participant.
- It focuses heavily on the exploration of ambivalence concerning behavior change using reflective listening.

**B. How Motivational Interviewing Informs DPC**

The following key principles of motivational interviewing guide the conduct of eCROPS-DPC:

- The physician and his/her patient become partners in the latter's health care;
- Motivational interviewing views patients as possessing the necessary capacities to alter their behavior;
- Motivational interviewing has respect for the patient's autonomy;

- Motivation to change is elicited from the patient, and not imposed from outside;
- It is the patient's task — not the physician's — to articulate and resolve his/her ambivalence;
- Direct persuasion is not an effective method for resolving ambivalence.
- The counseling style should generally be calm and eliciting.

Characteristics of a motivational interviewing style DO include:

- helping the patient to develop and verbalize arguments for change increases the likelihood of change;
- helping the patient when ready to develop a specific change plan also increases the likelihood of change;
- seeking to understand the patient's frame of reference, particularly through reflective listening;
- expressing acceptance and affirmation;
- eliciting and selectively reinforcing the participant's own self-motivational statements, expressions of problem recognition, concerns, desire and intention to change, and ability to change;
- monitoring the participant's degree of readiness to change, and ensuring that resistance is not generated by jumping ahead of the participant;
- affirming the patient's freedom of choice and self-direction.

Characteristics of a motivational interviewing style DO NOT include:

- arguing that the patient has a problem and needs to change;
- offering direct advice or prescribing solutions to the problem without actively encouraging the participant to make his/her own choices;
- using an authoritative/expert stance, leaving the participant in a passive role;
- doing most of the talking;
- behaving in a punitive or coercive manner.
