# Supplementary material for: A Smart Web Aid for Preventing Diabetes in Rural China: Preliminary Findings and Lessons
Source: J Med Internet Res. 2014 Apr 1;16(4):e98. doi: 10.2196/jmir.3228 (PMC4004141; doi:10.2196/jmir.3228)
Supplement: Supplementary file 2 [file jmir_v16i4e98_app2.pdf]

Appendix 2 Main functions of smart web aid for preventing diabetes (SWAP-DM2)

A: System configuration

a1: Enables setting/changing login information and assigning user authority.

a2: Enables entering/changing provider information (name, address, contacts etc.).

a3: Enables creating/maintaining a database of local diabetes-related referral service providers.

a4: Allows authorized users to enable/disable part of the optional functions.

a5: Enables authorized users to reset relevant default values in the system so as to suit local context.

a6: Enables individual doctor to select/adjust person-computer interface, e.g., color, font-size.

B: Appointment making

b1: Displays a minimum data set to facilitate registering necessary patient information.

b2: Generates unique reference number and provides a calendar to facilitate appointment making.

b3: Proposes standard operation procedures (SOP) and tips encouraging patient to seek services.

b4: Displays follow-up visits due in the forthcoming week or user defined time period.

C: Rapid integrated assessment and test

c1: Displays a short questionnaire to facilitate quick assessment of diabetes risks with the patient.

c2: Generates a risk score accordingly and helps classify the patient as high/low risk patient.

c3: Provides SOP/tips on encouraging high risk patient to receive glucose test.

c4: Provides SOP and tips on performing glucose test and notifying test result.

D: Counseling initial behavior changes

d1: Displays a flowchart of steps for counseling initial diabetes-related behavior changes.

d2: Provides SOP and tips on discussing risks and harms of elevated glucose status with patients.

d3: Provides tailored instruments for assessing specific patient's diabetes-related risk behaviors.

d4: Summarizes findings of the above assessment and generates a list of risk behaviors.

d5: Provides tips on alerting patient of his/her behavior risks and urgency to change.

d6: Provides tips/worksheet to facilitate selection of most important/feasible objective behavior(s).

d7: Provides tailored SOP/tips on motivating patient to enact set objective behavior(s).

d8: Provides tailored SOP/tips on counseling harms of target risk behaviors.

d9: Provides tailored SOP/tips on counseling benefits of objective behaviors.

d10: Provides SOP/tips counseling avoidance of potential dis-benefits of objective behaviors.

d11: Provides SOP/tips anticipating/overcoming potential barriers in implementing behavior change.

d12: Generates and displays follow up visit and referral service needs.

d13: Displays tips/worksheets to facilitate follow up visit and referral arrangements.

d14: Prints tailored take-away-leaflet facilitating behavior change and follow-up/referral services.

E: Counseling continuous behavior improvement

e1: Provides easy retrieval of previous prevention service records for a given client.

e2: Generates summary and "tree view" of past diabetes prevention of the patient under concern.

e3: Classifies the patient and leads to relevant branches of diabetes intervention workflow.

e4: Provides tailored instrument for reviewing previous lifestyle modification efforts by selected patient.

e5: Summarizes main progress made by the patient under concern.

e6: Provides SOP/tips on leveraging continued efforts on progress already made by the patient under concern.

e7: Identifies main problem(s) or areas to improve for the patients under concern.

e8: Provides tailored SOP/tips on solving the problem(s) identified.

e9: Provides tailored tips/instruments on performing complementary lifestyle assessment.

e10: Provides tailored tips/worksheets on setting complementary objective behaviors.

e11: Provides tailored tips/worksheets on motivating complementary objective behaviors.

e12: Provides visualized aid for calculating calorie intake and consumption and plans balanced diet/activity.

e13: Displays tips/worksheets to facilitate follow up visit and referral arrangements.

e14: Prints tailored "take away leaflet" to facilitate behavior change and follow up and referral

	services.
**F: Data management and utilization**	
f1: Provides easy means for real-time data entry and database upgrading.	f6: Provides easy means for retrieving specific past prevention case stored in the system.
f2: Provides an easy worksheet for entering and validating patient's test and measurement results.	f7: Displays diagrams showing status/trends in selected outcome/behavior indicators for selected patient(s).
f3: Provides aspects and easy means for doctors to retrieve past intervention case records.	f8: Displays diagrams showing motivation status/trends of selected patient practicing selected behavior.
f4: Produces aggregate reports of stored prevention cases grouped by doctor-selected indicators.	
f5: Produces and transmits routine diabetes prevention case reports requested by local CDC. (9.3)	f9: Provides tips and aspects for deriving approaches to tackle difficult objectives or patient.
	f10: Provides tips and aspects on planning forthcoming prevention sessions for a specific patient.
**G: Education and skills development**	
g1: Provides doctor-friendly and systematic slides regarding diabetes prevention.	g6: Proposes materials for specific doctor to learn upon his/her previous knowledge/skills assessment.
g2: Provides a systematic e-book on delivering effective diabetes prevention and case management.	g7: Provides easy means for doctors to refer common terminologies in relation to diabetes prevention.
g3: Provides a computerized tool for doctors to assess diabetes prevention knowledge/skills.	g8: Provides easy means for doctors to refer common diabetes-related indicators/figures/doses.
g4: Generates a visual report of overall and domains specific scores upon the above assessment.	g9: Provides easy means for doctors to mark and record comments on stored study materials.
g5: Depicts strong and weak knowledge/skills areas to inform doctor's future improvement plans.	g10: Provides easy means for doctors to add his/her own study materials to the web-based materials bank.
