# Supplementary material for: A Smart Web Aid for Preventing Diabetes in Rural China: Preliminary Findings and Lessons
Source: J Med Internet Res. 2014 Apr 1;16(4):e98. doi: 10.2196/jmir.3228 (PMC4004141; doi:10.2196/jmir.3228)
Supplement: Supplementary file 3 [file jmir_v16i4e98_app3.pdf]

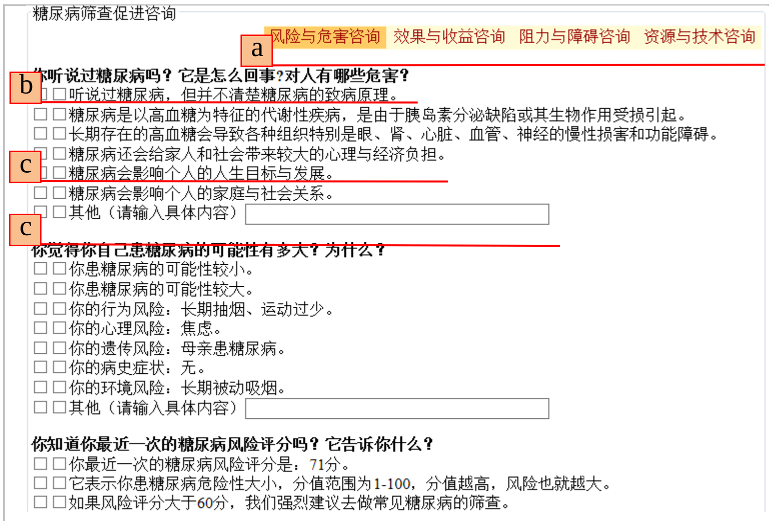

P1 Standard operation procedure (SOP) for counseling glucose test

a) Proposed SOP steps for counseling diabetes screening, including counseling risk and harms of diabetes, counseling effectiveness and benefits of glucose test, counseling dis-benefits and barriers of the test, providing resources and help for the test.

b) Suggested discussion questions.

c) Check boxes for doctor to record or remind the sample proposed key point listed on the left.

d) Text box for entering patient or doctor added new points.

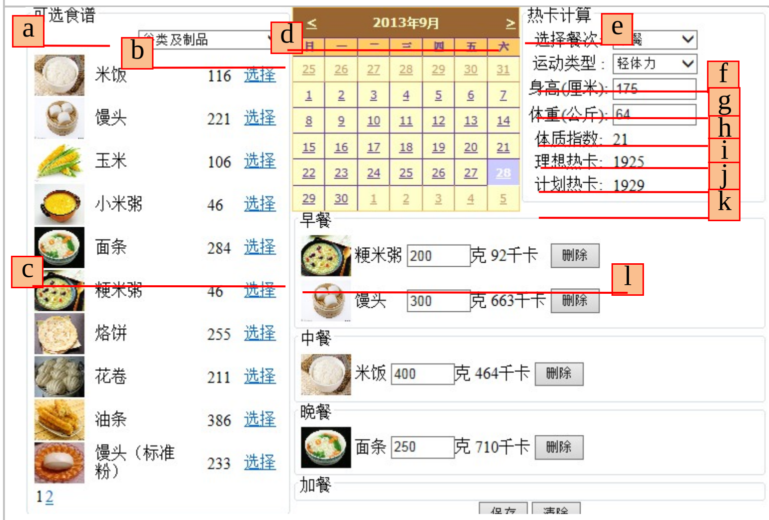

P2 Food calorie reference and diet planning work sheet

a) Commonly available foods.

b) Dropdown list of food categories.

c) Example food and estimated unit calorie, e.g., noodles estimated with 284 calorie per gram.

d) Calendar for selecting a specific date to plan diet.

e) Calorie calculator.

f) Activity level, e.g., light.

g) Height, e.g., 175 mm.

h) Body weight, e.g., 64 kg.

i) Body mass index, e.g., 21.

j) Ideal calorie intake, e.g., 1925.

k) Planned calorie intake, e.g.,1929

l) Selected food for breakfast, September 28, 2013.

Appendix 3 Sample application webpages of smart web aid for prevention (SWAP-DM2) against diabetes
