# Supplementary material for: A Smart Web Aid for Preventing Diabetes in Rural China: Preliminary Findings and Lessons
Source: J Med Internet Res. 2014 Apr 1;16(4):e98. doi: 10.2196/jmir.3228 (PMC4004141; doi:10.2196/jmir.3228)
Supplement: Supplementary file 4 [file jmir_v16i4e98_app4.pdf]

Appendix 4 Exit survey for smart web aid for preventing diabetes (SWAP-DM2) assessment

Q1 What are the harms of diabetes and pre-diabetes?

[ ] It leads to lesions in the eye, kidney and heart etc.
[ ] It affects long-term objectives and development.
[ ] It affects family and social relationships.
[ ] It induces psychological and economic burdens.

Q2 What are the harms of imbalanced diet?

[ ] It leads to overweight or obesity.
[ ] It causes hypertension.
[ ] It leads to cerebral-cardio-vascular diseases.
[ ] It induces diabetes.
[ ] It leads to cancer.

Q3 What are the harms of inadequate physical activity?

[ ] It leads to overweight or obesity.
[ ] It causes hypertension.
[ ] It leads to cerebral-cardio-vascular diseases.
[ ] It induces diabetes.
[ ] It leads to cancer.
[ ] It reduces body immunity.

Q4 What have you been regularly practicing for controlling your blood glucose in the past month?

[ ] Reduce calorie intake.
[ ] Increase vegetable intake.
[ ] Increase leisure-time exercises.

Q5 Please select a value from 0 to 10 to indicate your confidence for practicing each of the following behaviors.

[ ] Modifying diet.
[ ] Increasing physical activities.
[ ] Refusing snacks.
[ ] Engaging relatives in diabetes prevention.

Q6 Please select a value from 0 to 10 to indicate your satisfaction toward each of the following service aspects.

[ ] Overall service provided.
[ ] Service techniques used.

[ ] The doctor’s responsiveness.
